# Clinical and economic outcomes of myasthenia gravis and myasthenic crisis when treated with PLEX and IVIG

**DOI:** 10.3389/fneur.2025.1713388

**Published:** 2025-11-20

**Authors:** Makenzie Maroney, Tabitha Diehl, Eli Blaney, Saif Zurob, Abubakar Tauseef, Mohsin Mirza, Ali Bin Abdul Jabbar

**Affiliations:** 1 Department of Medicine, Creighton University School of Medicine, Omaha, NE, United States; 2 Department of Internal Medicine, Creighton University School of Medicine, Omaha, NE, United States

**Keywords:** myasthenia gravis, myasthenic crisis, intravenous immunoglobulin, plasmapheresis, mortality, length of stay, total hospital charge

## Abstract

**Introduction:**

Myasthenia gravis is an autoimmune disorder characterized by muscle weakness, with myasthenic crisis representing a life-threatening exacerbation requiring respiratory support. Acute treatment options include plasmapheresis (PLEX) and intravenous immunoglobulin (IVIG). Prior studies have reported mixed results regarding the relative effectiveness of these therapies, although recent evidence suggests that PLEX may provide faster and more noticeable clinical improvement. The objective of this study was to evaluate differences in clinical and economic outcomes among U.S. patients hospitalized for myasthenia gravis or crisis who received PLEX, IVIG, both, or neither treatment.

**Methods:**

Myasthenia gravis and crisis admissions from 2017 to 2022 were collected using the National Inpatient Sample. Admissions were categorized by treatment: IVIG, PLEX, both, or neither. To minimize selection bias, admissions were propensity-matched for IVIG and PLEX. Differences in mortality were evaluated using logistic regression models. Length of stay and inflation-adjusted total charge were assessed using linear regression models.

**Results:**

Most admissions received neither PLEX nor IVIG. Compared to treatment with neither, treatment with IVIG, PLEX, or both were not associated with significant differences in mortality; however, they were linked to longer hospital stays and higher total charge. A direct comparison of PLEX vs. IVIG revealed similar mortality and total charges, although length of stay was longer for PLEX. Older age was associated with increased mortality.

**Discussion:**

Although PLEX has been advocated as first-line therapy due to rapid clinical improvement, national data do not clearly indicate a superior treatment option, with similar mortality and total charges observed across therapies.

## 1 Introduction

Myasthenia gravis (MG) is an acquired, antibody-mediated autoimmune disease affecting the postsynaptic membrane of the neuromuscular junction. Antibodies against nicotinic acetylcholine receptors (AChR) degrade receptors and cause a failure in neuromuscular transmission, leading to muscle weakness (1). The most common comorbidities of MG include thyroid disease, systemic lupus erythematosus, and rheumatoid arthritis (2). In 2021, the prevalence of MG in the US was 37 per 100,000 persons, and this number continues to rise each year (3).

Myasthenic crisis is the most life-threatening manifestation of MG. It is characterized by requiring invasive or non-invasive ventilation due to respiratory muscle or bulbar weakness, causing upper airway collapse. Approximately 15%−20% of patients with MG will experience a myasthenic crisis, with an annual incidence of approximately 2.5% among all MG patients. Although the first crisis typically occurs between 8 and 12 months after diagnosis (4), a myasthenic crisis can be the initial presentation of MG in 18%−28% of cases (4).

There are four primary treatment modalities for MG: anticholinesterase agents to preserve acetylcholine in the synaptic spaces, surgical thymectomy to remove AChR-specific CD4+ T cells, immunosuppression, and short-term immunotherapies that include plasma exchange (PLEX) and intravenous immunoglobulin (IVIG) (5). PLEX, or plasmapheresis, removes circulating antibodies, including those against the AChR. PLEX is most effective in circumstances where short-term benefit is required, including patients in myasthenic crisis, patients before and after surgeries, and in patients with inadequate response to medications or thymectomy. IVIG binds to and blocks the effects of antibodies, including those against AChR and has similar indications to PLEX (6, 7). The choice between these therapies often depends on patient-specific factors and comorbidities; for instance, PLEX is contraindicated in patients with sepsis, while IVIG is avoided in those with hypercoagulable states, renal failure, or immunoglobulin hypersensitivity (6).

Evidence comparing PLEX and IVIG remains mixed. As of 2023, only four controlled trials have directly compared the two treatments. Two of these can be compared as they used the Quantitative Myasthenia Gravis Score for disease severity (QMGS) to measure improvement (8). One study of 84 patients with moderate to severe MG reported similar response rates: 69% for IVIG and 65% for PLEX. Additionally, both treatments were well-tolerated and provided similar duration of benefit (9). The second study of 40 patients with late-onset MG compared PLEX, IVIG, and immunoadsorption, which removes autoantibodies semi-selectively. The results indicated that all modalities decreased QMGS scores and ameliorated symptoms. However, plasmapheresis and immunoadsorption showed greater reductions in QMGS, shorter hospital stays, and less need for ventilatory support. Beyond controlled trials, several review studies (4, 10, 11) and a 2016 international consensus (6) have indicated that plasmapheresis leads to faster and more noticeable clinical improvement.

Due to the few controlled trials, previous studies have advocated for the continued monitoring of large databases as a means to compare the efficacy and safety of IVIG and PLEX when treating MG and myasthenic crisis (12). We used the National Inpatient Sample (NIS) to assess the impact of PLEX and IVIG on mortality and resource utilization among admissions with a primary diagnosis of MG or myasthenic crisis.

## 2 Methods

### 2.1 Data source and ethics statement

The National Inpatient Sample (NIS) database provided hospital data from 2017–2022. The NIS is part of the Healthcare Cost and Utilization Project (HCUP) and is the largest publicly available all-payer inpatient healthcare database in the United States; the NIS, weighted, represents approximately 35 million hospitalizations nationally (13). Data from 2023 onward were not yet available at the time of collection. This research is exempt from institutional review board approval, as it only used publicly available, de-identified data adherent to 45 CFR §46.

### 2.2 Patients

All hospitalizations for myasthenia gravis and myasthenic crisis in patients ≥18 years old were included. MG-specific hospitalizations were identified using the International Classification of Diseases-10 Clinical Modification (ICD-10-CM) codes G70.00 as a primary diagnosis. Myasthenic crisis was identified for admissions with a primary diagnosis using ICD-10-CM code G70.01 or G70.00 plus one of the following: J96.00, J96.01, J96.02, or interventions coded by 0BH17EZ, 0BH13EZ, 0BH18EZ, 5A09357, 5A09457, 5A09557, 5A1935Z, 5A1945Z, 5A1955Z, or Z99.11. Hospitalizations in which the patient was diagnosed with transient neonatal myasthenia gravis (ICD-10-CM: P94.0), Lambert-Eaton (ICD-10-CM: G70.80, G70.81), chronic inflammatory demyelinating polyneuritis (ICD-10-CM: G61.81), critical illness polyneuropathy (ICD-10-CM: G62.81), critical illness myopathy (ICD-10-CM: G72.81), polyneuropathy in diseases classified elsewhere (ICD-10-CM: G63), acute paralytic poliomyelitis, unspecified (ICD-10-CM: A80.30), acute poliomyelitis, unspecified (ICD-10-CM: A80.9), acute non-paralytic poliomyelitis (ICD-10-CM: A80.4), acute poliomyelitis (ICD-10-CM: A80), acute transverse myelitis in demyelinating disease of central nervous system (ICD-10-CM: G37.3), poisoning by, adverse effect of and underdosing of other and unspecified drugs, medicaments and biological substances (ICD-10-CM: T50.9), alcohol dependence with intoxication, unspecified (ICD-10-CM: F10.229) were excluded. Hospitalizations for Myasthenia Gravis and Myasthenia Gravis crisis subgroups were differentiated based on the primary diagnosis and intubation and ventilation status.

### 2.3 Exposure

Admissions were grouped into those that received PLEX, IVIG, both, or neither. PLEX was defined by either of the following ICD-10 procedure codes: 6A550Z3 for a single plasmapheresis or 6A551Z3 for multiple plasmapheresis. IVIG was defined as having any of the following ICD-10-CM codes: 30233S1, 30243S1, XW133D7.

### 2.4 Outcomes

The primary study outcome was in-hospital mortality. Length of stay and total hospital charge were evaluated as secondary outcomes. Hospital charge was inflation-adjusted to mid-year 2024 US dollars (14).

### 2.5 Statistical analysis

Continuous variables are presented as mean and standard error, compared using Kruskal-Wallis test, whereas categorical variables are presented as percent, compared using the Pearson's *X*^2^ with Rao-Scott adjustment. Differences in all-cause in-hospital mortality were evaluated using logistic regression models. Hospital length of stay and inflation-adjusted total hospital charge were evaluated using linear regression models.

To minimize bias from treatment selection and disease heterogeneity, we used propensity score matching. Propensity scores were estimated by a subclassification model using PLEX, IVIG, and both, as the outcome and matching for the following demographic, administrative, and clinical determinants of treatment: age, biological sex, race, payer, hospital location-teaching study, hospital region, median household income in zip code, hospital bed size, weekend admission, elective admission, Elixhauser sum, chronic obstructive pulmonary disease, cardiac arrhythmia, valvular disease, pulmonary circulation disease, peripheral vascular disease, uncomplicated hypertension, paralysis, other neurological disorders, uncomplicated diabetes, complicated diabetes, hypothyroidism, renal failure and disease, liver disease, peptic ulcer, lymphoma, metastatic cancer, solid tumor without metastasis, rheumatoid arthritis, obesity, weight loss, fluid and electrolyte disorders, deficiency anemias, drug abuse, psychoses, depression, complicated hypertension, COVID-19, thymoma, systemic lupus erythematosus, acute renal failure, chronic kidney disease, alcohol abuse, chronic blood loss anemias, autoimmune thyroiditis, unspecified kidney failure, acquired immune deficiency syndrome, endotracheal intubation, CPAP or BiPAP, disseminated intravascular coagulation, hereditary factor VIII deficiency, Von Willebrand disease, hereditary factor XI deficiency, hereditary deficiency of other clotting factors, hemorrhagic disorder due to circulating anticoagulants, acquired coagulation factor deficiency, primary thrombophilia, other thrombophilia, other specified coagulation defects, coagulation defects unspecified, qualitative platelet defects, immune thrombocytopenic purpura, other primary thrombocytopenia, secondary thrombocytopenia, thrombocytopenia unspecified, adverse effects of immunoglobulin initial encounter, sepsis, and severe sepsis. The propensity score was subclassified into six groups using the MatchIt package in R (15, 16). The quality of the match was evaluated by comparing distributions of the propensity scores (histograms, cumulative distribution functions) as well as standardized differences for individual covariates used to estimate the propensity score. The decision to retain non-linear forms was determined using a likelihood ratio test (17). All analyses were conducted using R version 4.4.2, accounted for the NIS sampling design, and were weighted to estimate national-level effects. Two-tailed *p* < 0.05 was defined to indicate statistical significance.

## 3 Results

A total of 58,200 admissions were identified to have a primary diagnosis of MG or myasthenic crisis between 2017 and 2022 (Table 1). There were no significant differences in the mean age (*p* = 0.084) or proportions by gender (*p* = 0.074) between the treatment groups. There were significantly more admissions involving white patients than other races (72% white, *p* = 0.001), and the rate of Medicare usage was greater than Medicaid or private insurance (59% Medicare, *p* < 0.001). Admissions involving administration of both IVIG and PLEX were more likely to have patients in the highest income quartile (32% of admissions for both vs. 22% overall, *p* < 0.001). There were significantly more admissions that were non-elective (87%, *p* < 0.001), in the southern region (44%, *p* < 0.001), and in large hospitals (60%, *p* < 0.001).

**Table 1:** Baseline demographic characteristics.

**Demographic characteristics**	**Overall *n* = 58,200*^a^***	**IVIG *n* = 9,530*^a^***	**PLEX *n* = 9,835*^a^***	**Both *n* = 825*^a^***	**Neither *n* = 38,010*^a^***	***p-value^b^***
**Age, years**	61.9 ± 0.2	61.6 ± 0.5	61.8 ± 0.4	64.3 ± 1.3	62.0 ± 0.2	0.084
**Biological sex**						0.074
Female	31,595 (54%)	5,345 (56%)	5,425 (55%)	390 (47%)	20,435 (54%)	
Male	26,600 (46%)	4,185 (44%)	4,410 (45%)	435 (53%)	17,570 (46%)	
**Race**						0.001
White	40,460 (72%)	6,475 (70%)	7,200 (76%)	620 (79%)	26,165 (72%)	
Black	7,965 (14%)	1,420 (15%)	1,230 (13%)	50 (6.4%)	5,265 (14%)	
Hispanic	5,415 (9.7%)	995 (11%)	695 (7.3%)	75 (9.6%)	3,650 (10%)	
Asian or Pacific Islander	1,170 (2.1%)	175 (1.9%)	180 (1.9%)	10 (1.3%)	805 (2.2%)	
Native American	305 (0.5%)	45 (0.5%)	80 (0.8%)	10 (1.3%)	170 (0.5%)	
Other	650 (1.2%)	115 (1.2%)	125 (1.3%)	15 (1.9%)	395 (1.1%)	
**Insurance status**						<0.001
Medicare	32,800 (59%)	5,285 (58%)	5,605 (60%)	495 (61%)	21,415 (59%)	
Medicaid	6,570 (12%)	1,220 (13%)	790 (8.5%)	50 (6.2%)	4,510 (13%)	
Private insurance	15,925 (29%)	2,595 (29%)	2,925 (31%)	265 (33%)	10,140 (28%)	
**Median household income in zip code**						<0.001
0–25th percentile	15,835 (28%)	2,400 (26%)	2,770 (28%)	160 (20%)	10,505 (28%)	
26–50th percentile	14,700 (26%)	2,290 (24%)	2,415 (25%)	225 (28%)	9,770 (26%)	
51–75th percentile	14,250 (25%)	2,310 (25%)	2,430 (25%)	160 (20%)	9,350 (25%)	
76–100th percentile	12,555 (22%)	2,390 (25%)	2,105 (22%)	260 (32%)	7,800 (21%)	
**Elective admission**						<0.001
Non-elective	50,650 (87%)	9,075 (95%)	9,025 (92%)	800 (97%)	31,750 (84%)	
Elective	7,500 (13%)	450 (4.7%)	800 (8.1%)	25 (3.0%)	6,225 (16%)	
**Hospital region**						<0.001
Northeast	10,025 (17%)	2,140 (22%)	1,515 (15%)	165 (20%)	6,205 (16%)	
Midwest or North Central	11,840 (20%)	1,445 (15%)	2,145 (22%)	145 (18%)	8,105 (21%)	
South	25,485 (44%)	4,115 (43%)	4,760 (48%)	360 (44%)	16,250 (43%)	
West	10,850 (19%)	1,830 (19%)	1,415 (14%)	155 (19%)	7,450 (20%)	
**Hospital bed size**						<0.001
Small	8,205 (14%)	1,350 (14%)	800 (8.1%)	85 (10%)	5,970 (16%)	
Medium	15,045 (26%)	2,375 (25%)	2,050 (21%)	145 (18%)	10,475 (28%)	
Large	34,950 (60%)	5,805 (61%)	6,985 (71%)	595 (72%)	21,565 (57%)	

^a^Mean ± SE; n (%).

^b^Design-based Kruskal Wallis test; Pearson's *X*^2^: Rao & Scott adjustment.

Admissions with a primary diagnosis of myasthenic crisis contributed approximately 75% of admissions, whereas MG accounted for approximately 25% of the admissions. Thirty-eight thousand and ten admissions did not receive IVIG or PLEX (Table 2). For those with a primary diagnosis of MG, approximately 86% did not receive either IVIG or PLEX. Comparatively, for those with a primary diagnosis of myasthenic crisis, only approximately 59% of admissions did not receive intervention with IVIG or PLEX.

**Table 2:** Baseline clinical characteristics.

**Clinical characteristics**	**Overall *n* = 58,200*^a^***	**IVIG *n* = 9,530*^a^***	**PLEX *n* = 9,835*^a^***	**Both *n* = 825*^a^***	**Neither *n* = 38,010*^a^***	***p-value^b^***
**Primary diagnosis and respiratory support**						
Myasthenia gravis	14,170 (24.3%)	1,310 (13.7%)	680 (6.9%)	20 (2.4%)	12,160 (32.0%)	—
Myasthenic crisis	44,030 (75.7%)	8,220 (86.3%)	9,155 (93.1%)	805 (97.6%)	25,850 (68.0%)	—
Endotracheal intubation	5,315 (9.1%)	785 (8.2%)	1,570 (16%)	295 (36%)	2,665 (7.0%)	<0.001
CPAP or BiPAP	4,770 (8.2%)	980 (10%)	1,175 (12%)	235 (28%)	2,380 (6.3%)	<0.001
**Relative contraindications to PLEX**						
Sepsis	1,520 (2.6%)	220 (2.3%)	495 (5.0%)	75 (9.1%)	730 (1.9%)	<0.001
Severe sepsis	755 (1.3%)	100 (1.0%)	280 (2.8%)	30 (3.6%)	345 (0.9%)	<0.001
**Relative contraindications to IVIG**						
Disseminated intravascular coagulation	165 (0.3%)	10 (0.1%)	110 (1.1%)	20 (2.4%)	25 (< 0.1%)	<0.001
Primary thrombophilia	470 (0.8%)	75 (0.8%)	90 (0.9%)	15 (1.8%)	290 (0.8%)	0.484
Other thrombophilia	350 (0.6%)	70 (0.7%)	85 (0.9%)	10 (1.2%)	185 (0.5%)	0.136
Other specified coagulation defects	245 (0.4%)	5 (< 0.1%)	165 (1.7%)	15 (1.8%)	60 (0.2%)	<0.001
Coagulation defect unspecified	420 (0.7%)	15 (0.2%)	210 (2.1%)	20 (2.4%)	175 (0.5%)	<0.001
Adverse effect of IVIG initial encounter	445 (0.8%)	180 (1.9%)	45 (0.5%)	20 (2.4%)	200 (0.5%)	<0.001
Acute renal failure	5,575 (9.6%)	725 (7.6%)	1,255 (13%)	125 (15%)	3,470 (9.1%)	<0.001
Renal failure and disease	6,575 (11%)	885 (9.3%)	1,195 (12%)	95 (12%)	4,400 (12%)	0.026
Unspecified kidney failure	30 (< 0.1%)	15 (0.2%)	10 (0.1%)	0 (0%)	5 (< 0.1%)	0.061
**Common comorbidities**						
Thymoma	305 (0.5%)	65 (0.7%)	85 (0.9%)	5 (0.6%)	150 (0.4%)	0.044
Autoimmune thyroiditis	770 (1.3%)	155 (1.6%)	130 (1.3%)	5 (0.6%)	480 (1.3%)	0.531
Hypothyroidism	12,065 (21%)	2,035 (21%)	2,155 (22%)	175 (21%)	7,700 (20%)	0.383
SLE	955 (1.6%)	165 (1.7%)	210 (2.1%)	0 (0%)	580 (1.5%)	0.103
Rheumatoid arthritis	3,390 (5.8%)	545 (5.7%)	640 (6.5%)	20 (2.4%)	2,185 (5.7%)	0.164
**Other comorbidities**						
Elixhauser sum	3.27 ± 0.02	3.14 ± 0.05	3.71 ± 0.05	3.84 ± 0.17	3.18 ± 0.03	<0.001
Other neurological disorders	6,485 (11%)	995 (10%)	1,140 (12%)	115 (14%)	4,235 (11%)	0.467
COVID-19	745 (1.3%)	230 (2.4%)	110 (1.1%)	20 (2.4%)	385 (1.0%)	<0.001
AIDS	60 (0.1%)	10 (0.1%)	15 (0.2%)	0 (0%)	35 (< 0.1%)	0.867

^a^Mean ± SE; n (%).

^b^Design-based Kruskal Wallis test; Pearson's *X*^2^: Rao & Scott adjustment.

For those who were treated, 9,530 admissions received IVIG, 9,835 received PLEX, and 825 received both (Table 2). Of the admissions with MG who received the specified treatments, approximately 64% received IVIG, 35% received PLEX, and 1% received both. Of the admissions with myasthenic crisis who received the specified treatments, approximately 45% received IVIG, 50% received PLEX, and 4% received both.

Additionally, there were a few notable significant differences in baseline clinical characteristics, including Elixhauser sum (both 3.84 ± 0.17; neither 3.18 ± 0.03; *p* < 0.001), sepsis (IVIG 220 (2.3%); both 75 (9.1%); overall 1,520 (2.6%); *p* < 0.001), disseminated intravascular coagulation (PLEX 110 (1.1%); both 20 (2.4%); overall 165 (0.3%); *p* < 0.001), and acute renal disease (PLEX 1,255 (13%); both 125 (15%); overall 5,575 (9.6%); *p* < 0.001) (Table 2).

### 3.1 Overall clinical and economic outcomes

When comparing the average outcomes between groups, the data suggest that the treatment of MG and myasthenic crisis with both IVIG and PLEX was associated with the highest mortality rate (6.7%), longest length of stay (17.2 ± 0.7 days), and greatest total charges ($341,006 ± 20,261). Treatment with PLEX was associated with the next highest mortality (3.5%), length of stay (10.7 ± 0.2 days), and charge ($181,510 ± 4,633). IVIG alone had the same mortality as admissions treated without IVIG or PLEX (2.2%), but was associated with a longer stay (6.5 ± 0.1 vs. 6.1 ± 0.1 days) and higher charges ($156,016 ± 3,290 vs. $118,267 ± 1,827).

### 3.2 Propensity-matched clinical and economic outcomes compared to neither treatment

The outcomes for MG and myasthenic crisis admissions treated with IVIG, PLEX, or both compared to neither treatment, with respective propensity matching, are displayed in Table 3. Propensity score matching was performed to adjust for differences in demographic and administrative variables, as well as clinical determinants of treatment choice.

**Table 3:** Multivariate regression analysis of PLEX and IVIG treatment outcomes compared to neither treatment with respective propensity matching.

**Treatment & Propensity Matched Group**	**Myasthenia gravis (*****n*** = **14,360)**						**Myasthenic crisis(*****n*** = **43,840)**					
	**IVIG**	***p***	**PLEX**	***p***	**Both**	***p***	**IVIG**	***p***	**PLEX**	***p***	**Both**	***p***
Mortality (OR)	2.64	0.24	4.66	0.073	0.00	_	1.15	0.58	0.68	0.13	0.96	0.95
Length of stay, mean difference (days)	3.4	<0.001	1.1	<0.001	12	<0.001	2.4	<0.001	−0.30	0.066	6.7	<0.001
Total charge, mean difference ($)	44,125	<0.001	43,926	<0.001	199,519	<0.001	18,005	<0.001	20,935	<0.001	127,159	<0.001

Treatment of MG with IVIG, PLEX, and both was not associated with a significant change in mortality. However, the treatments were associated with an increase in length of stay and total charge when compared to neither treatment.

Treatment of myasthenic crisis with IVIG, PLEX, or both was not associated with a significant difference in mortality when compared to receiving neither treatment. Treatment with PLEX and both was associated with an increase in length of stay when compared to treatment with neither PLEX nor IVIG; however, treatment with IVIG was not associated with a difference in length of stay compared to receiving neither treatment. The total charge was increased with the use of either and both treatment modalities compared to neither PLEX nor IVIG.

### 3.3 Propensity-matched clinical and economic outcomes of PLEX compared to IVIG

The outcomes for MG and myasthenic crisis admissions treated with PLEX compared to IVIG, with propensity matching to PLEX, IVIG, and both, are displayed in Table 4. Propensity score matching adjusted for demographic, administrative, and clinical determinants of treatment selection. Treatment with PLEX was not associated with a significant difference in mortality or total hospital charge compared to treatment with IVIG for both MG and myasthenic crisis admissions. However, PLEX was associated with an increased length of stay (mean difference = 2.7 days, *p* < 0.001).

**Table 4:** Multivariate regression analysis of PLEX outcomes compared to IVIG outcomes with propensity matching.

**Propensity matched group**	**Myasthenia gravis (*****n*** = **14,360)**		**Myasthenic crisis (*****n*** = **43,840)**	
	**Estimate (reference** = **IVIG)**	***p***	**Estimate (reference** = **IVIG)**	***p***
Mortality (OR)	1.33	0.33	1.46	0.23
Length of stay, mean difference (days)	2.2	<0.001	2.8	<0.001
Total charge, mean difference ($)	1,207	0.93	−1,773	0.72

Additional analysis showed that increasing age was significantly associated with higher mortality in the propensity-matched analysis. Overall, the odds of mortality for each additional year of age was 1.09 (OR: 1.09, 95% CI 1.06 to 1.12, *p* < 0.001). In the MG group, the odds of mortality were 1.19 for each additional year of age (OR: 1.19, 95% CI 1.08 to 1.30, *p* < 0.001). In the myasthenic crisis group, the odds of mortality were 1.09 for each additional year of age (OR: 1.09, 95% 1.06 to 1.12, *p* < 0.001).

Furthermore, with propensity matching for any intervention, the following clinical characteristics were significantly associated with higher mortality overall: Elixhauser sum (OR: 1.80, 95% CI: 1.04 to 3.12, *p* = 0.036), endotracheal intubation (OR: 2.69, 95% CI: 1.80 to 4.01, *p* < 0.001), CPAP or BIPAP (OR: 1.89, 95% CI: 1.20 to 2.89, *p* = 0.006), acute renal failure (OR: 1.83, 95% CI: 1.21 to 2.79, *p* = 0.005), severe sepsis (OR: 2.91, 95% CI: 1.16 to 7.29, *p* = 0.023), COVID 19 (OR: 6.26, 95% CI: 2.91 to 12.5, *p* < 0.001), and AIDS (OR: 65.0, 95% CI: 7.84 to 540, *p* < 0.001). Notably, all of the listed clinical characteristics, except Elixhauser sum, had a similar impact on mortality for the myasthenic crisis group. However, the odds of mortality for the MG group were not significantly impacted by any of the clinical characteristics.

## 4 Discussion

In this study, hospital admissions for MG and myasthenic crisis treated with IVIG, PLEX, or both were not associated with a significant change in mortality when compared to admissions that were not treated with either therapy. However, use of these treatments was associated with an increase in length of stay and total hospital charges when compared to treatment with neither. The comparisons made between treatment modalities were rendered more reliable through bias reduction achieved by propensity score matching.

To better interpret the collected analysis, it is essential to recognize that guidelines from American and European institutions recommend the use of PLEX in cases of severe myasthenia gravis, including myasthenic crisis (18). PLEX is likely favored in more severe cases because of higher odds of any improvement (11) and faster clinical recovery (19). This clinically translates to superior short-term symptom improvement (11), improved ventilatory status, and earlier extubation (19). Furthermore, it is recommended that PLEX be prioritized when combining PLEX and IVIG, as a synergistic effect can be achieved only if PLEX precedes IVIG. If PLEX is performed after IVIG, it removes the administered IgG, thereby nullifying IVIG's effect (8). Evidence from our study supporting the recommendation that PLEX is used in more severe cases is reflected in the significantly higher percentage of intubated admissions among patients treated with PLEX or both modalities. Additionally, a significantly greater percentage of admissions who were septic or severely septic were treated with PLEX or both modalities, despite sepsis being a relative contraindication to treatment with PLEX.

Overall, mortality did not significantly differ between treatment groups. The absence of a significant difference in mortality between patients treated with IVIG and PLEX is consistent with prior studies demonstrating no clear difference in efficacy among these treatments for moderate-to-severe MG (9, 12). There are two major considerations to account for when considering the efficacy of these treatments in terms of mortality. First, the treatment groups did not start equally, as evidenced by significant differences in clinical characteristics shown in Table 2. Despite our attempt to identify and adjust for these differences, some factors, like severity, could not be directly identified by ICD-10 codes and therefore could not be adjusted for. This makes it challenging to directly compare the efficacy of treatments, with the known potential that the PLEX group may have more severe cases because of current guidelines. Secondly, while PLEX and IVIG should be considered first-line therapies for myasthenic crisis (4), patients who received neither may have been managed with other modalities, including anticholinesterase agents, surgical thymectomy, and immunosuppressive therapy (5). As such, the efficacies of PLEX and IVIG are not being compared to a specific control. Therefore, while our findings align with existing evidence suggesting comparable outcomes between PLEX and IVIG, the absence of a mortality difference should be interpreted with caution, as it may be influenced by variations in disease heterogeneity, treatment selection, and adjunctive treatment strategies.

In general, treatment with PLEX and/or IVIG increased length of stay significantly compared to treatment with neither. An indication for PLEX and IVIG is when treatment with medication or thymectomy was inadequate. As such, there is potential that other interventions were employed before PLEX and IVIG, thereby lengthening the hospital stay. The only non-significant difference in length of stay occurred when myasthenic crisis treatment with PLEX was compared to treatment with neither. This finding may reflect that PLEX, which is directly indicated for myasthenic crisis, was initiated more promptly and used before other treatment strategies were tried. When directly comparing PLEX to IVIG, our results suggest that admissions receiving PLEX had a length of stay that was approximately 2 to 3 days longer than those receiving IVIG. This aligns with previous data suggesting that the average duration of the treatment of an MG exacerbation with IVIG is 2 to 5 days, compared with PLEX, which requires five sessions spread over seven to 10 days (4). However, previous studies have suggested PLEX provides faster clinical improvement (4, 10, 11). Clinical improvement with IVIG is typically seen between 4 days and 1 week after initiation. Improvement from PLEX is seen within a couple of days or after two to three treatment sessions (4, 20). The discrepancy between faster clinical improvement and longer hospitalization when using PLEX is likely multifaceted, with differences in treatment protocol being a primary reason. Additionally, a comprehensive review explained that IVIG-receiving patients tended to have fewer complications and were significantly less likely to require treatment discontinuation. The review also reinforced the current guidelines by suggesting that IVIG administration is more convenient to use in milder cases, while plasmapheresis is often reserved for more severe cases that are typically associated with longer hospitalization (11). Given the recommendation to use PLEX in more severe cases, the observed differences in hospitalization duration may reflect treatment selection and disease severity rather than a true difference in treatment efficacy. Therefore, the statistically significant difference may not be clinically applicable.

The general trend for cost analysis suggested that utilizing PLEX or IVIG accrued greater charges than not using either treatment modality. The utilization of these treatments, either alone or in combination, increased charges by anywhere from $20,000 to over $200,000. A previous study that performed an itemized comparative cost-minimization analysis of PLEX vs. IVIG for myasthenic crisis found that, for both treatment modalities, the most significant contributors to cost were ICU costs and immunoglobulin or albumin and exchange costs, respectively (21). The study also reported that, compared with PLEX, IVIG use saved patients an average of $22,326 (21). They further identified that the most significant contributors to the higher cost of PLEX are the extended ICU and hospital stays. However, in our analysis, there was no significant difference in charges accrued from the use of PLEX vs. IVIG. The comparable charges between PLEX and IVIG treatment regimens found in our study may reflect the individualized quantity and duration of treatment. More specifically, the number of plasma exchanges required, the IVIG dosing, and the number of days in the ICU were three variables that significantly affected the cost comparison (21). For example, since IVIG is administered based on patient weight, higher patient mass can necessitate larger total doses (21). In addition to variations in treatment regimen, the lack of difference in charge across modalities may reflect adjustments introduced by propensity matching, which limit the impact of clinical characteristics. Overall, our findings suggest that, despite previous reports of cost savings with IVIG, charges may be more similar than previously estimated once treatment variations and patient characteristics are accounted for.

It is important to continue to assess the risks and benefits of IVIG and PLEX because of the prevalence of MG and myasthenic crisis. Analysis of NIS data from 2010 to 2019 determined that hospitalization rates of patients with MG and myasthenic crisis increased at a statistically significant annualized rate, while mortality rates remained stable (22). As such, it is crucial we determine the most efficacious and efficient modality of treatment to protect healthcare resources and better serve patients. Additionally, since the previous NIS data analysis has shown stable mortality rates, future studies should also assess the role of alternative medical and surgical interventions to better understand how to optimize outcomes for patients with MG and myasthenic crisis.

Another reason continuing this comparative analysis is essential is that IVIG and PLEX are used to treat other acute neurological conditions, including Guillain-Barré syndrome, an acute inflammatory demyelinating polyradiculoneuropathy (23). Therefore, the relevance and benefits of comparing the efficacy of the two treatments extend beyond patients with MG.

### 4.1 Limitations

Our study has several important limitations inherent to the use of the NIS database. First, the observational data provided by the NIS prevents any causal relationship between treatment modality and outcomes from being made. Second, coding inaccuracies and misclassification are possible when relying on ICD codes, which may affect the identification of a primary diagnosis. We attempted to account for this by including both ICD codes for myasthenic crisis and MG with respiratory distress in our myasthenic crisis group; however, this attempt was not foolproof. Third, the NIS lacks detailed clinical variables that could influence treatment selection and outcomes, such as disease severity scores, neurologic examination findings, antibody status, and comorbidities. We attempted to limit this bias through propensity score matching, specifically with clinical factors that may have influenced treatment selection, including intensity of respiratory support and certain relative contraindications to treatment. However, not all clinical factors that impact treatment indication were included in the analysis. Lastly, important longitudinal information that could influence treatment selection and outcomes is left out as well, including outpatient medication use, prior treatments, and readmission. Notably, we were unable to exclude patients who were historically treated with immune checkpoint inhibitor therapy or those who experienced adverse effects related to this therapy. These specific exclusions would be relevant as this immunotherapy can cause neurologic toxicities, specifically myositis and MG (24). As such, this therapy can cause disease courses that differ from typical MG or lead to misdiagnosis of MG. Future studies are needed to validate and expand on these findings and further focus on how individual factors influence treatment selection and outcomes.

### 4.2 Conclusion

This analysis of recent NIS data did not provide sufficient evidence to establish superiority of PLEX or IVIG in the treatment of MG or myasthenic crisis as both modalities demonstrated similar mortality and charges, and the significantly longer length of stay associated with PLEX may have been due to the impact of clinical characteristics on treatment selection.

## Data Availability

Publicly available datasets were analyzed in this study. This data can be found here: National Inpatient Sample—Healthcare Cost and Utilization Project at https://hcup-us.ahrq.gov/nisoverview.jsp.
